# Rapid diagnostic test negative *Plasmodium falciparum* malaria in a traveller returning from Ethiopia

**DOI:** 10.1186/s12936-021-03678-2

**Published:** 2021-03-12

**Authors:** Stefan Schlabe, Ingrid Reiter-Owona, Tamara Nordmann, Ramona Dolscheid-Pommerich, Egbert Tannich, Achim Hoerauf, Jürgen Rockstroh

**Affiliations:** 1 Department of Internal Medicine I, University Hospital of Bonn, Venusberg Campus 1, Building 26, 53127 Bonn, Germany; 2 Institute for Medical Microbiology, Immunology and Parasitology (IMMIP), University Hospital of Bonn, Bonn, Germany; 3 Institute of Clinical Chemistry and Clinical Pharmacology, University Hospital Bonn, Bonn, Germany; 4 National Reference Centre for Tropical Pathogens, Bernhard Nocht-Institute for Tropical Medicine (BNITM), Hamburg, Germany; 5 German Centre of Infection Research, Partner Site Bonn-Cologne, Bonn, Germany

**Keywords:** Malaria, RDT, HRP2, Ethiopia

## Abstract

**Background:**

*Plasmodium falciparum* strains with mutations/deletions of the genes encoding the histidine-rich proteins 2/3 (*pfhrp2/3*) have emerged during the last 10 years leading to false-negative results in HRP2-based rapid diagnostic tests (RDTs). This can lead to unrecognized infections in individuals and to setbacks in malaria control in endemic countries where RDTs are the backbone of malaria diagnostics and control.

**Case description:**

Here the detection of a *pfhrp2/3*-negative *P. falciparum* infection acquired in Ethiopia by a 63-year old female traveller is presented. After onset of symptoms during travel, she was first tested negative for malaria, most probably by RDT, at a local hospital in Harar, Ethiopia*.* Falciparum malaria was finally diagnosed microscopically upon her return to Germany, over 4 weeks after infection. At a parasite density of approximately 5387 parasites/µl, two different high-quality RDTs: Palutop + 4 OPTIMA, NADAL^R^Malaria PF/pan Ag 4 Species, did not respond at their respective *P. falciparum* test lines. *pfhrp2/3* deletion was confirmed by multiplex-PCR. The patient recovered after a complete course of atovaquone and proguanil. According to the travel route, malaria was acquired most likely in the Awash region, Central Ethiopia. This is the first case of imported *P. falciparum* with confirmed *pfhrp2/3* deletion from Ethiopia.

**Conclusion:**

HRP2-negative *P. falciparum* strains may not be recognized by the presently available HRP2-based RDTs. When malaria is suspected, confirmation by microscopy and/or qPCR is necessary in order to detect falciparum malaria, which requires immediate treatment. This case of imported *P. falciparum*, non-reactive to HRP2-based RDT, possibly underlines the necessity for standardized, nationwide investigations in Ethiopia and should alert clinicians from non-endemic countries to the possibility of false-negative RDT results which may increase in returning travellers with potentially life-threatening infections.

**Supplementary Information:**

The online version contains supplementary material available at 10.1186/s12936-021-03678-2.

## Background

Rapid diagnostic tests (RDTs) are lateral flow immunochromatographic assays, which detect *Plasmodium* antigens present in blood containing infected erythrocytes. RDTs, which focus on the detection of *Plasmodium falciparum* commonly use the histidine-rich protein 2 (Pf-HRP2) as major target antigen. Tests with other targets, such as *P. falciparum*-specific or pan-parasite lactate dehydrogenase (Pf-pLDH, pLDH) and aldolase, are available but their use is associated with lower sensitivity and a reduced stability at high temperatures [1].

A major component of the HRP2-based RDT is a monoclonal anti-HRP2 mouse antibody. This antibody shows cross-reactivity with the HRP3 antigen, a protein of quite similar composition, which is also present in developing stages of *P. falciparum*, albeit at much lower concentration [2].

*Plasmodium falciparum* isolates with deletion of *pfhrp2* and *pfhrp3* were first described in the Amazonas region of Peru in samples collected in 2005 [3]. In the following years, an increasing number of reports showed that *P. falciparum* isolates with single (*pfhrp2-* or *pfhrp3-*) or double (*pfhrp2-* and *pfhrp3-*) deletion are present in many countries and on different continents [4]. There is concern that a potentially life-threatening infection with *P. falciparum* will not be recognized by HRP2-based RDTs, especially in countries or regions where the *P. falciparum* transmission rate is low but where a high number of strains with deletions circulate.

It is estimated that in sub-Saharan African countries RDTs fail to detect *P. falciparum* in 1.4 to 100% of cases [5]. In Eritrea, a rate of 80% false negative RDT results (SD Bioline) occurred in 50 microscopically confirmed *P. falciparum* samples, which had been collected in two different regional hospitals [6]. This situation could increase the risk not only for the endemic population but also for travellers to become infected with deleted strains. Even high quality RDTs would not perform adequately for the diagnosis of falciparum malaria during and after travel. In this communication, a case is presented of a HRP2-negative, symptomatic *P. falciparum* infection acquired by a traveller in Ethiopia, and the impact of negative RDT results on malaria case management in non-endemic countries is discussed.

### Case description

A 63-years-old patient presented in the Walk-in-Clinic for Infectious Diseases of the University Hospital in Bonn, Germany, 10 days after returning from a 6-week trip to Ethiopia. The patient and her accompanying husband had received pre-travel advice for repellent administration and bed-net use and were provided with an on-demand dose of anti-malarials (atovaquone/proguanil). Vaccination against hepatitis A/B and yellow fever was performed prior to travel. She had never been diagnosed with malaria before and she reported two short stays in malaria-endemic regions before, in Okavango Delta in 2007 (with chemoprophylaxis) and in Amazon rainforest, Peru, in 2014 (with stand-by medication). There was no longer residency in an endemic country before.

**Fig. 1:**
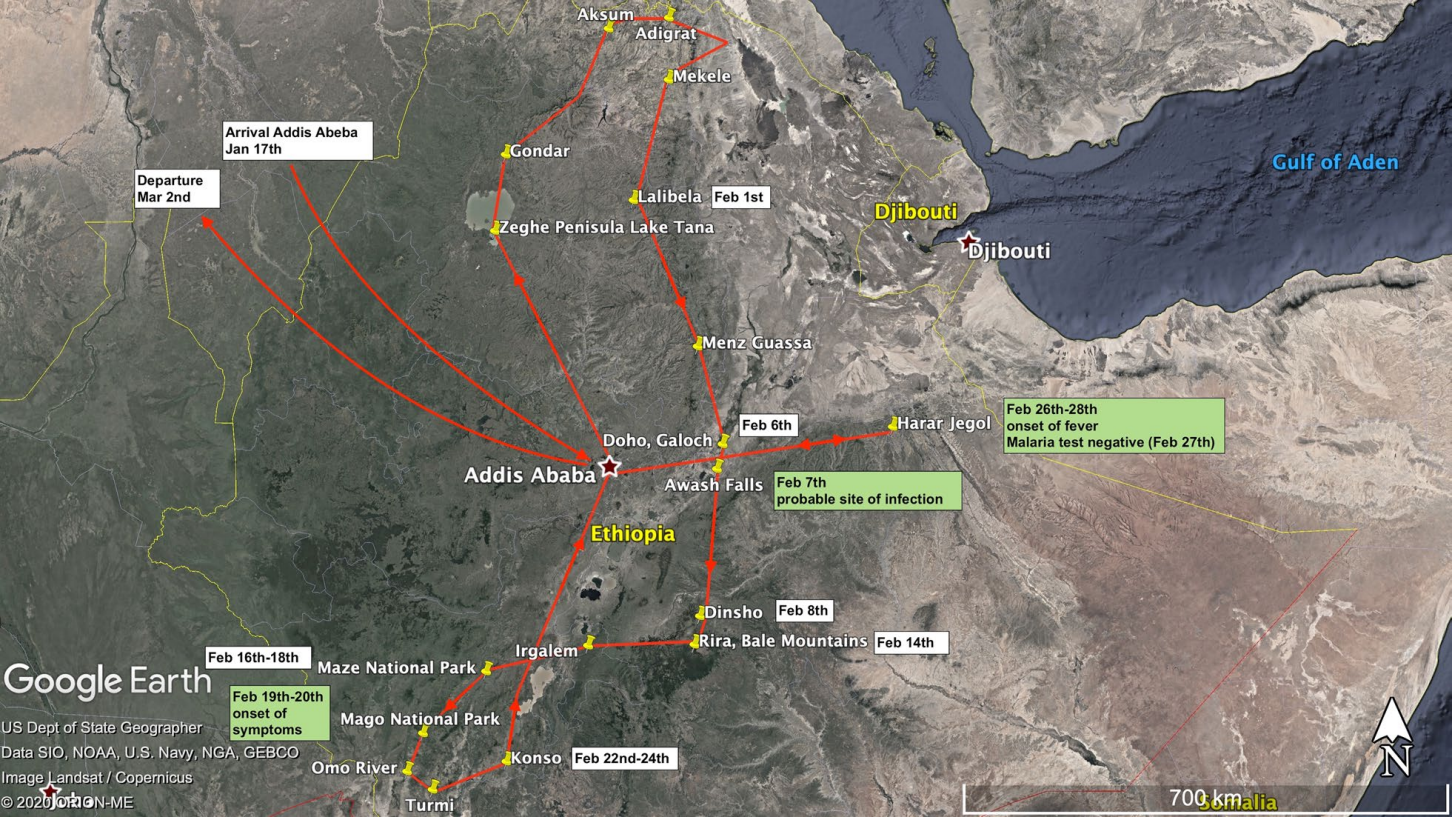
Travel route of the patient in Ethiopia, with timeline and labelling of the main events (Courtesy of the patient and her husband)

The couple arrived in Addis Ababa on 17 January, 2020. Most of the trip took place in the highlands. Data of the travel route and the events were provided by the patient and her husband, (Fig. 1). On 7 and 8 February they crossed a malaria-endemic region next to the Awash Falls (400–500 m above sea level), where the patient registered mosquito bites despite repellent use. After arrival at the national parks in the south (south of Maze National Park), the patient experienced fatigue, retrospectively regarded as first sign of illness (19 February, day 11 after possible infection). Subsequently, the symptoms aggravated with diarrhoea, loss of appetite and general malaise. Her husband noticed tachypnoea while sleeping. Because of fever and night sweats she presented to a local hospital in Harar (27 February, day 19 after possible infection), where malaria was excluded (the diagnostic method used, RDT or microscopy, is not reported), and therefore stand-by atovaquone/proguanil was not taken. Other antibiotics with partial activity against malaria, such as doxycycline, were also not taken. After returning to Germany on 2 March, the patient presented to her general practitioner with ongoing illness. Tests for influenza, Sars-CoV2 and schistosomiasis were performed but not for malaria. As her condition continued to deteriorate, she was referred to the Walk-in-Clinic for Infectious Diseases on 12 March (day 33 after possible infection) for malaria testing and further evaluation.

On presentation, the patient was feverish with compensated blood pressure and heart rate (BP 110 over 70 mm Hg, HR 92 per minute, 38.1 °C, oxygen saturation 96%, body weight 60 kg, height 170 cm). Examinations revealed a newly described systolic murmur and splenomegaly. Initial blood count showed pancytopenia (leukopenia 2.97 G/L, Hb 10.5 g/dl and thrombocytopenia 80 G/L). C-reactive protein was only slightly elevated to 11.22 mg/L. Lactate dehydrogenase was elevated (360 U/L), sodium was reduced to 132 mmol/L. Other laboratory parameters, such as creatinine, transaminases and bilirubin, were in normal range. As the main differential diagnosis, malaria tests were performed (both RDT and microscopy), blood cultures, X-ray of the thorax and tests for dengue and chikungunya due to a current outbreak situation in Eastern Africa.

**Fig. 2:**
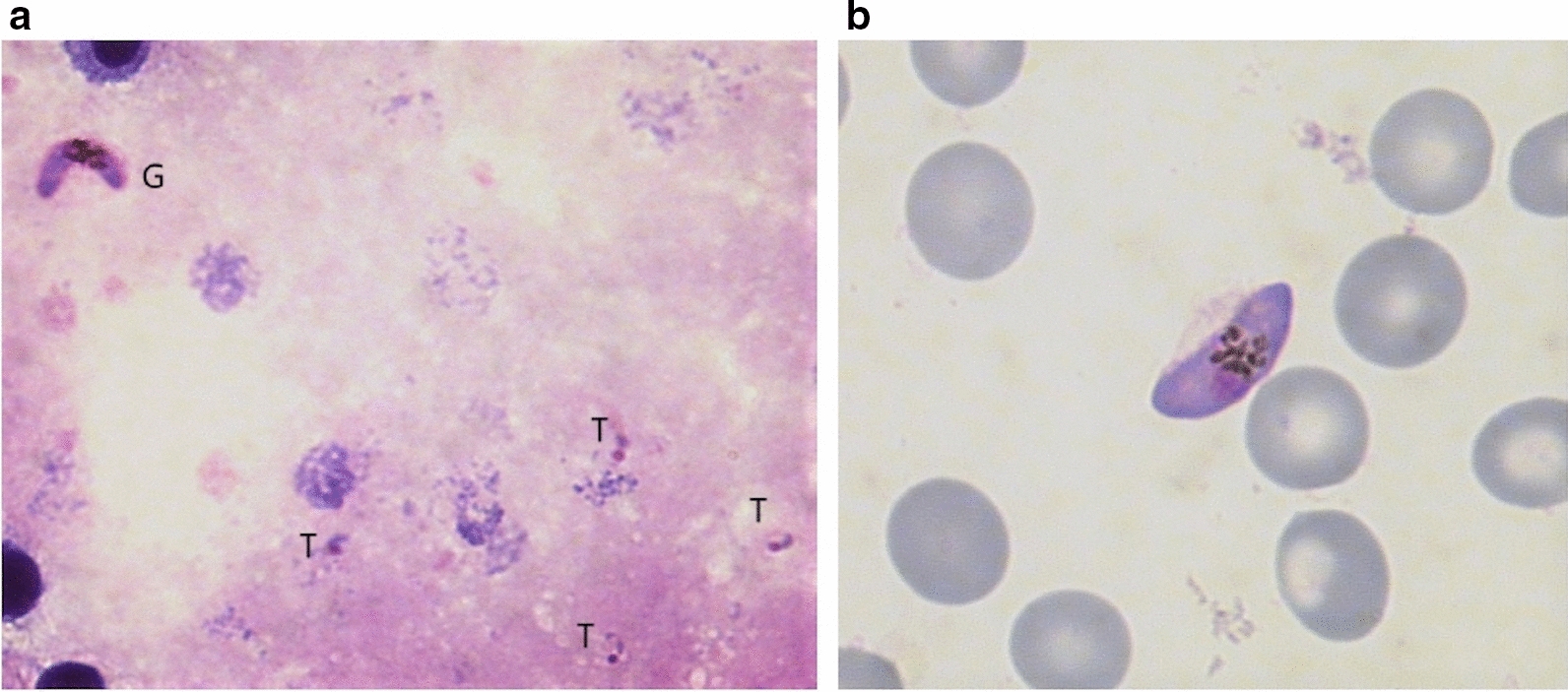
Blood sample taken at presentation in the Walk-in-Clinic, day 34 after possible infection. **a** Giemsa-stained thick smear. *Plasmodium falciparum*, T = Trophozoite, G = Gametocyte. **b** Giemsa-stained thin smea. *Plasmodium falciparum*, G = Gametocyte

*Plasmodium falciparum* infection was demonstrated microscopically in Giemsa-stained thin and thick blood smears. Typical ring forms and crescent-shaped gametocytes were visible (Fig. 2a/b). Parasite density was determined as 5,387 parasites per microlitre. Two different HRP2-based RDTs (Palutop + 4 OPTIMA, Biosynex, France; NADAL^R^Malaria PF/pan Ag 4 Species, nal von minden GmbH, Germany) were used. Palutop + 4 is a RDT with four bands (Pf-HRP2, *Plasmodium vivax*-specific LDH Pv-pLDH and pan-*Plasmodium*-specific LDH). The limit of detection for *P. falciparum* is 100 parasites per microlitre at “Pan” and “Pf” bands. The limit of detection for *P. vivax* is 200 parasites per microlitre at “Pan” and “Pv” bands. NADAL RDT test has three bands (Pf-HRP2 and pan-*Plasmodium* LDH). The sensitivity was tested against thick blood smear test: *P. falciparum* detection: sensitivity 92.6%, specificity 100%. “Pan” malaria detection: sensitivity 95%, specificity 100%.

**Fig. 3:**
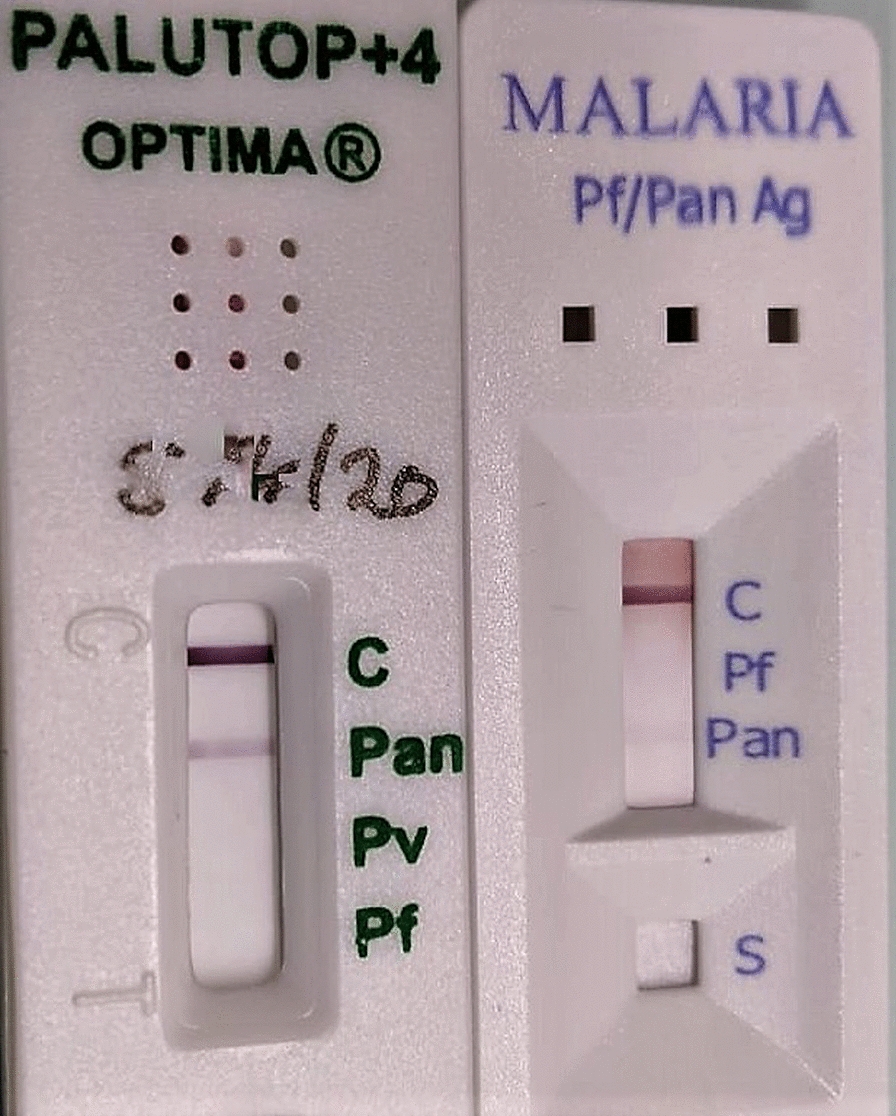
Negative *Plasmodium falciparum* (Pf) result but positive to weak positive “Pan” result in two different quality-controlled rapid diagnostic tests. RDTs are pre-coated with: left: Palutop + 4 OPTIMA (Biosynex, France): Pan line = pLDH (common), ma. Pv line = pLDH (*P. vivax*), mab. Pf line = HRP2 protein (*P. falciparum*), mab right: NADAL^R^Malaria PF/pan Ag 4 Species (nal v. minden, Germ.) Pan line = pLDH (common), mab Pf line = HRP2 protein (*P. falciparum*), polyclonal antibody. mab = monoclonal antibody

Both tests showed no reaction at the “Pf” test line, but moderate (Palutop + 4 OPTIMA) to weak positive (NADAL^R^Malaria PF/pan Ag 4 species) reaction at the “Pan” test line (Fig. 3). Due to these discrepant results, an aliquot of the blood sample was analysed at the National Reference Centre for Tropical Pathogens (BNITM, Hamburg), where PCR proved infection with *P. falciparum* and excluded other *Plasmodium* species, such as *P. vivax*, *Plasmodium ovale*, *Plasmodium malariae* or *Plasmodium knowlesi*. Moreover, a molecular analysis by multiplex PCR according to [7] performed at the BNITM confirmed a deletion in both *pfhrp2* and *pfhrp3* (Additional file 1: Fig. S1).

All other evaluations were without pathological finding. Blood cultures remained sterile. X-ray of the thorax showed no pulmonary oedema or infiltrates. Echocardiography revealed an ejection fraction of 60.1% without signs of valve vitium or endocarditis.

A complete course of atovaquone and proguanil (Malarone^R^) was administered. After 3 days in hospital, the patient could be discharged with improved condition. At follow-up one week later, both RDTs were negative not only at the “Pf” line but also at the “Pan” line. In addition, thick blood films were negative for *P. falciparum* trophozoites.

## Discussion

*Plasmodium. falciparum* RDTs are point-of-care tests to detect malaria antigens (Pf-HRP2 or pLDH) in the patient`s blood by an immunochromatographic assay. False-negative RDTs can arise for different reasons, among them very low (< 250 parasites per microlitre) or high parasitaemia, and dysfunctional tests due to non-functioning antibodies or test equipment after improperly stored test kits. Moreover, variants or lack of HRP2 proteins in the parasite result in false-negative results for *P. falciparum* [4]. HRP2-based rapid tests are a mainstay of point-of-care testing for malaria in endemic regions and support diagnosis in returning travellers. In 2014, RDTs were used for 71% of malaria diagnoses in sub-Saharan Africa [8]. In settings where neither microscopy nor PCR is available, malaria diagnosis relies exclusively on RDTs. Since parasites with diversity in *pfhrp2* and parasites lacking HRP2 are emerging in malaria-endemic regions, there is much concern that *P. falciparum* infections remain under-diagnosed and therefore not treated in time [1].

After the first published observation of *pfhrp2-*negative strains in the Amazon region of Peru in 2010 with frequencies of up to 41% [3], parasites with *pfhrp2* deletion have since been described worldwide [4]. South American countries include Bolivia, Brazil, Colombia, Ecuador, French Guiana, Guyana, Honduras, and Suriname (summarized by Gendrot et al. [4]). The highest frequencies in Africa were published for Ghana (36%) [9], Rwanda (23%) [10], Eritrea (9,7%)[11], and Democratic Republic of Congo (6.4%) [12]. HRP2-negative parasites were described in Asia, albeit in low frequencies: in India [13] and along the border between China, Thailand and Myanmar [14]. In small pockets, frequency can be very high, e.g., in Odisha, India [15]. Very recently, *pfhrp2*-negative parasites were described in the Greater Mekong Sub-region in a frequency of 9.4% [16]. To date, 29 countries have reported *pfhrp2*- and/or *pfhrp3* strains, as reported in the World Health Organization (WHO) Malaria Threat Map [8, 17], with Ethiopia being the most recently added country. *Plasmodium falciparum* isolates with deletion of the *pfhrp2* and *pfhrp3* genes were identified in Ethiopia, shown by a study from the Adama Malaria Diagnostic Centre in Central Ethiopia [18], but the extent and geographic distribution of these strains is not yet known.

A study in northwestern Ethiopia showed a reduced sensitivity of RDTs (70.8%) when compared to PCR results. This finding may be associated with RDT failures in this region but still lacks confirmation at genome level [19] as the PCR has been repeatedly proven to be the diagnostic method of the highest sensitivity. Girma et al*.* [20] also described deletions in samples collected in a highly endemic region of southwest Ethiopia. Due to a low parasite density in asymptomatic-infected individuals, which in most cases was beyond the detection level of the different RDTs applied, only a few samples could be evaluated at genomic level and showed evidence of RDT failure.

Adding the findings from this case where a traveller was most probably infected in Central Ethiopia close to the Awash Falls, an area known to be endemic for malaria, suggests a wider spread problem in this country than anticipated. Bordering Eritrea, Ethiopia can be considered at high-risk, with strong implications for local malaria RDT testing and control measurements.

In this traveller’s sample, *P. falciparum* was confirmed by microscopy and PCR and parasitaemia was counted according to WHO standards [1]. The same sample did not react at the “Pf” test lines of two quality-controlled *pfhrp*2-detecting RDTs. A parasite concentration of 5387 per microlitre is far above the diagnostic cut-off level of both of the applied RDTs (*P. falciparum* limit of detection: NADAL^R^Malaria PF/pan Ag 4 species = 100 parasites per microlitre at “Pf” and “Pan” test lines; NADAL^R^Malaria PF/pan Ag 4 species = 250 parasites per microlitrel at “Pf” test line). The positive reactions at the “Pan” test line (= common pLDH) of both RDTs could confirm malaria but not define the species involved. In Ethiopia, other species such as *P. vivax* amount to approximately 40% of all cases, and low-scale infections with *P. ovale* and *P. malariae* have been reported [19, 21]. In this case, species other than *P. falciparum* were excluded by PCR.

The detection of gametocytes in the blood smears confirmed the assumed date of infection (7 or 8 February) retrospectively. At presentation, the patient had been suffering from falciparum malaria for approximately 4 weeks. Fortunately, parasitaemia remained moderate and she did not develop severe malaria, according to WHO criteria. Unrecognized uncomplicated falciparum malaria can result in higher parasitaemia and progress to severe malaria in 10% of cases [22]. It is noteworthy that the missed diagnosis in a European woman without endemic immunologic background resulted in a non-severe course of malaria at > 30 days after probable infection. A first report that *pfhrp*-deleted strains have reduced fitness and produce a lower parasite density in humans [12] might explain the clinical findings but need confirmation. However, the patient suffered from a pronounced malaise.

At the time of early presentation at a local hospital (approximately 19 days after infection), parasitaemia might have been sufficient for microscopic diagnostic in a non-immune patient. In Ethiopia, a combined RDT (CareStart™ Malaria HRP2/pLDH COMBO Test, Access Bio Inc, USA) which detects *P. falciparum*-specific HRP2 and pan-*Plasmodium* LDH (pLDH) is widely used, according to the WHO’s country profile database [23]. This test has excellent agreement with light microscopy [24]. Theoretically, it can detect *pfhrp2*-negative parasites via pLDH (Pan line) as shown in the laboratory at > 30 days post infection. However, the test’s sensitivity for pLDH is low during recent *P. falciparum* single infection with low parasitaemia. As a consequence, a complete negative result of the combination RDT is highly likely at the local setting. Advising the patient to repeat malaria testing within a few days would have been recommended. The available stand-by medication was not used, which is in accordance with recommendations of current guidelines of the German Society of Tropical Medicine and Global Health (DTG). An increasing number of cases, like the one reported here, would justify to recommending regular chemoprophylaxis instead of stand-by medication for travellers to countries where deletions in *pfhrp2* gene of *P. falciparum* are known. In this case, chemoprophylaxis would have avoided disease and a false negative malaria test.

Similar cases have occasionally been described in travellers, such as in a French traveller after a visit to Brazil in 2011 [25]. According to a recent French review, the overall rate of negative RDTs is very low in returning travellers and no case with *pfhrp2/3* deletions was detected [26]. This situation may change in the near future as a consequence of malaria eradication programmes which rely on HRP2 -detecting RDTs to recognize and treat *P. falciparum* cases, thus creating a selection pressure on strains with *pfhrp2* deletions [27]. WHO recommends that endemic countries switch to non-HRP2-based RDTs only at a threshold of 5% of non-reactivity [28].

HRP2-based RDTs combined with, for example pLDH, are valuable tools as part of routine clinical case management in non-malaria-endemic countries. At present, there is no combination rapid test that meets WHO criteria and which could be used to detect HRP2 and/or HRP3-deficient *P. falciparum* strains with certainty. Pan-LDH-only RDTs show promising results when tested against both HRP2 expressing and non-expressing *P. falciparum* panels [29]. According to several national guidelines, each RDT result needs confirmation by microscopy and/or qPCR, a strategy that will prevent cases with HRP2-deletion being overlooked. In the event of quality-controlled microscopy not being available, the WHO recommends that all malaria suspects in endemic regions should be treated presumptively with artemisinin-based combination therapy (ACT) [29]. There is no resistance connected to *P. falciparum* strains with *pfhrp2* deletion, only chloroquine resistance is described for *P. falciparum* strains with *pfhrp2* deletion (summarized by Cheng et al. [1]). The patient discussed here responded well to a standard atovaquone and proguanil therapy with complete parasite clearance and recovery.

## Conclusion

Reported here is the first case of a returning traveller who acquired a HRP2- negative *P. falciparum* strain in Ethiopia, most probably in the Awash region. This case illustrates that false-negative results by HRP2-based RDTs may lead to delayed diagnosis and treatment during and/or after travel. Travellers should be advised to ask for a microscopic blood examination when travelling in an area which is endemic for HRP2-negative *P. falciparum* strains. Clinicians in endemic and non-endemic countries should made aware of emerging HRP2-negative strains and the implications for RDT testing in *P. falciparum*-infected patients.

## Supplementary Information

**Additional file 1: Fig. S1.** Multiplex detection of *pfhrp2* and *pfhrp3* genes on the patient's sample (green) and a *P. falciparum* with known sequence with wild type *pfhrp2/3* (red) using deletions assay according to [7]. A pfhrp2 in patient and control sample. Amplification only in control; threshold cycle (CT) = 19.25; B *pfhrp3* in patient and control sample. Amplification only in control; CT 23.70; C internal control in patient and control sample. Detection in patient's sample (CT = 24.12) and control sample (CT = 18.68).

## Data Availability

Not applicable.
